# Supplementary material for: Iterative improvement in the automatic modular design of robot swarms
Source: PeerJ Comput Sci. 2020 Dec 7;6:e322. doi: 10.7717/peerj-cs.322 (PMC7924708; doi:10.7717/peerj-cs.322)
Supplement: Supplemental Information 3 [file peerj-cs-06-322-s003.zip › argos3/doc/api/standalone/a00414.html]

ARGoS: plugins/robots/e-puck/simulator/epuck\_entity.cpp File Reference


- Main Page
- Related Pages
- Namespaces
- Classes
- Files

- File List
- File Members

# plugins/robots/e-puck/simulator/epuck\_entity.cpp File Reference

`#include "epuck_entity.h"`  
`#include <argos3/core/utility/math/matrix/rotationmatrix3.h>`  
`#include <argos3/core/simulator/space/space.h>`  
`#include <argos3/core/simulator/entity/controllable_entity.h>`  
`#include <argos3/core/simulator/entity/embodied_entity.h>`  
`#include <argos3/plugins/simulator/entities/rab_equipped_entity.h>`  
`#include <argos3/plugins/simulator/entities/ground_sensor_equipped_entity.h>`  
`#include <argos3/plugins/simulator/entities/led_equipped_entity.h>`  
`#include <argos3/plugins/simulator/entities/light_sensor_equipped_entity.h>`  
`#include <argos3/plugins/simulator/entities/proximity_sensor_equipped_entity.h>`  

Include dependency graph for epuck\_entity.cpp:

Go to the source code of this file.

|  |  |
| --- | --- |
| Namespaces | |
| namespace | argos |

|  |  |
| --- | --- |
|  | The namespace containing all the ARGoS related code. |

| Defines | |
| #define | UPDATE(COMPONENT)   if(COMPONENT->IsEnabled()) COMPONENT->Update(); |
| Functions | |
|  | argos::REGISTER\_ENTITY (CEPuckEntity,"e-puck","Carlo Pinciroli [ilpincy@gmail.com]","1.0","The e-puck robot.","The e-puck is a open-hardware, extensible robot intended for education. In its\n""simplest form, it is a two-wheeled robot equipped with proximity sensors,\n""ground sensors, light sensors, a microphone, a frontal camera, and a ring of\n""red LEDs. More information is available at http://www.epuck.org\n\n""REQUIRED XML CONFIGURATION\n\n"" <arena ...>\n"" ...\n"" <e-puck id=\"eb0\">\n"" <body position=\"0.4,2.3,0.25\" orientation=\"45,90,0\" />\n"" <controller config=\"mycntrl\" />\n"" </e-puck>\n"" ...\n"" </arena>\n\n""The 'id' attribute is necessary and must be unique among the entities. If two\n""entities share the same id, initialization aborts.\n""The 'body/position' attribute specifies the position of the pucktom point of the\n""e-puck in the arena. When the robot is untranslated and unrotated, the\n""pucktom point is in the origin and it is defined as the middle point between\n""the two wheels on the XY plane and the lowest point of the robot on the Z\n""axis, that is the point where the wheels touch the floor. The attribute values\n""are in the X,Y,Z order.\n""The 'body/orientation' attribute specifies the orientation of the e-puck. All\n""rotations are performed with respect to the pucktom point. The order of the\n""angles is Z,Y,X, which means that the first number corresponds to the rotation\n""around the Z axis, the second around Y and the last around X. This reflects\n""the internal convention used in ARGoS, in which rotations are performed in\n""that order. Angles are expressed in degrees. When the robot is unrotated, it\n""is oriented along the X axis.\n""The 'controller/config' attribute is used to assign a controller to the\n""e-puck. The value of the attribute must be set to the id of a previously\n""defined controller. Controllers are defined in the <controllers> XML subtree.\n\n""OPTIONAL XML CONFIGURATION\n\n""You can set the emission range of the range-and-bearing system. By default, a\n""message sent by an e-puck can be received up to 80cm. By using the 'rab\_range'\n""attribute, you can change it to, i.e., 4m as follows:\n\n"" <arena ...>\n"" ...\n"" <e-puck id=\"eb0\" rab\_range=\"4\">\n"" <body position=\"0.4,2.3,0.25\" orientation=\"45,90,0\" />\n"" <controller config=\"mycntrl\" />\n"" </e-puck>\n"" ...\n"" </arena>\n\n""You can also set the data sent at each time step through the range-and-bearing""system. By default, a message sent by an e-puck is 2 bytes long. By using the""'rab\_data\_size' attribute, you can change it to, i.e., 20 bytes as follows:\n\n"" <arena ...>\n"" ...\n"" <e-puck id=\"eb0\" rab\_data\_size=\"20\">\n"" <body position=\"0.4,2.3,0.25\" orientation=\"45,90,0\" />\n"" <controller config=\"mycntrl\" />\n"" </e-puck>\n"" ...\n"" </arena>\n\n","Under development") |
|  | argos::REGISTER\_STANDARD\_SPACE\_OPERATIONS\_ON\_COMPOSABLE (CEPuckEntity) |

---

## Define Documentation

|  |  |  |  |  |  |
| --- | --- | --- | --- | --- | --- |
| #define UPDATE | ( | COMPONENT |  | ) | if(COMPONENT->IsEnabled()) COMPONENT->Update(); |

Definition at line 269 of file epuck\_entity.cpp.

---

Generated on 10 Jul 2018 for ARGoS by 
 1.6.1
